# Recurrent burner syndrome due to presumed cervical spine osteoblastoma in a collision sport athlete – a case report

**DOI:** 10.1186/1749-7221-2-13

**Published:** 2007-06-06

**Authors:** Ilan Elias, Michael A Pahl, Adam C Zoga, Maurice L Goins, Alexander R Vaccaro

**Affiliations:** 1 Department of Orthopaedic Surgery, Rothman Institute, Thomas Jefferson University Hospital, Philadelphia PA, USA; 2 Department of Radiology, Thomas Jefferson University Hospital, Philadelphia PA, USA; 3 Naval Medical Center San Diego, Spine Surgery, Department of Orthopaedic Surgery, San Diego, CA, USA

## Abstract

We present a case of a 35-year-old active rugby player presenting with a history of recurrent burner syndrome thought secondary to an osteoblastoma involving the posterior arch of the atlas. Radiographically, the lesion had features typical for a large osteoid osteoma or osteoblastoma, including osseous expansion, peripheral sclerosis and bony hypertrophy, internal lucency, and even suggestion of a central nidus. The patient subsequently underwent an en bloc resection of the posterior atlas via a standard posterior approach. The surgery revealed very good clinical results.

In this report, we will discuss in detail, the presentation, treatment, and return to play recommendations involving this patient.

## Background

Athletes frequently develop cervical radicular symptoms as a result of a blunt injury to the head or neck, particularly when participating in contact or collision sports such as american football, soccer, rugby, wrestling and others. Any athletic endeavor leading to a collision may cause abrupt cervical axial compression, flexion, or extension producing a neurapraxia of the exiting nerve roots or brachial plexus due to traction or direct compression. In this scenerio, athletes sometimes experience a burning pain, which radiates distal from the posterior neck region to the fingertips. This constellation of symptoms is often referred to as a burner syndrome or "stinger" [Table 1]. Burners are typically isolated transient events, but can sometimes become recurrent and may even develop to a chronic syndrome [1,2].

**Table 1:** Differential Diagnosis Radiculopathy versus Stinger

Radiculpathy	Stinger
Monoradicular	Polyradicular
hypersensitivity or numbness	immediate pain
sensory symptoms > motor symptoms	symptoms few minutes
difficult to localize	global transient weakness
tingling, dull, aching	weakness, tingling, burning

Multiple underlying morphological factors exist which have been associated with the incidence of cervical spinal injuries in athletics including congenital or developmental spinal stenoses, congenital fusions, or intervertebral disk herniations or degeneration [3,4].

Other developmental anomalies that may predispose to subsequent neural compressive injury include spina bifida, Langerhans cell histocytosis (eosinophilic granuloma), exostoses, fibrous dysplasia, and melorheostosis. Additionally, posttraumatic lesions causing osseous enlargement could similarly predispose to later injury.

However, to our knowledge, there have been no reports of a burner syndrome developing through a contact sport injury related to an underlying expansile cervical spine lesion.

We present a case of a rugby player with a unique clinical history of recurrent burners thought to be secondary to an osteoblastoma involving the posterior arch of the atlas. Initially, the condition was felt to most likely reflect previous trauma and a reparative osseous proliferation. After complete imaging evaluation, the lesion was felt to more likely reflect a developmental lesion with bony expansion narrowing the central canal.

In this report, we will detail the presentation, treatment, postsurgical outcome, and return to play recommendations for this patient.

## Case presentation

A 35-year-old active rugby player with a one-year history of multiple recurrent stingers or burners in his left upper extremity presented to the senior author for evaluation three weeks following his most recent episode. In that episode, while playing rugby, the patient was involved in a head on collision with another player. The subject was referred to our orthopaedic surgery spine clinic due to a positive L'Hermitte's sign. The patient reported a brief loss of consciousness and states he awoke with a "stiff neck". He also stated that he experienced a burning and tingling pain shooting down his left upper extremity into all five fingers. The pain worsened with activity and was non-dermatomal. His symptoms improved over the subsequent hour after the trauma, and had completely resolved after 48 hours later. He denied any loss of hand or fine finger dexterity or bowel or bladder dysfunction. He also denied any history of fever, chills, weight loss, night pain, nausea or vomiting. He did however admit to intermittent episodes of cervical neck pain, with exacerbation during neck movement, in the interval between the trauma and the office visit, which responded well to nonsteroidal anti-inflammatory medications (NSAIDs).

On physical examination, cervical range of motion was limited to 10 degrees of extension and 45 degrees of rotation with no restriction in active flexion or extension. There were no motor or sensory deficits. Reflexes were equal bilaterally, with no upper motor neuron signs noted. Provocative tests such as flexion, extension and Spurling's sign that were performed were negative or unrevealing.

Plain radiographic evaluation (AP, lateral, flexion, extension cervical radiographs) revealed a mild decrease in cervical lordosis on the neutral lateral view and a hypertrophied, blastic appearance to the posterior arch of the atlas.

**Figure 1:**
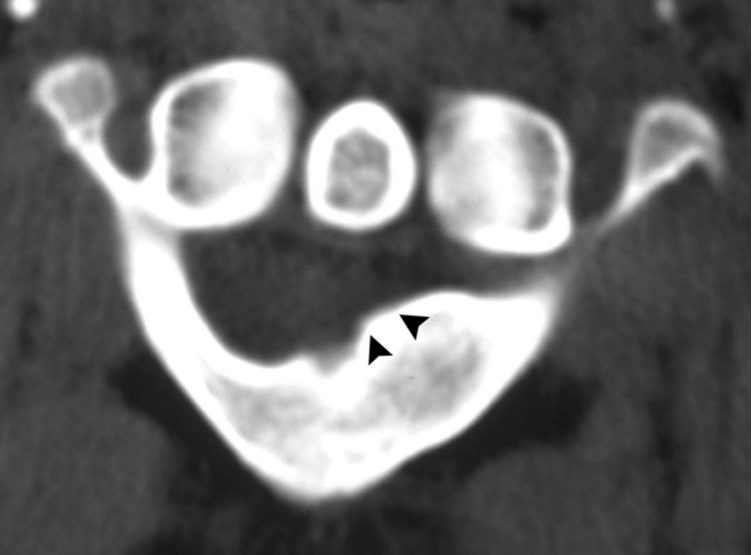
Fig 1 Axial (arrowheads) and Fig 2 sagittal CT demonstrate an expansile lesion (arrow) of the posterior arch of C1. It is contained within the cortex with no soft tissue extension. The bony margins appear smooth, homogeneous and sclerotic.

**Figure 2:**
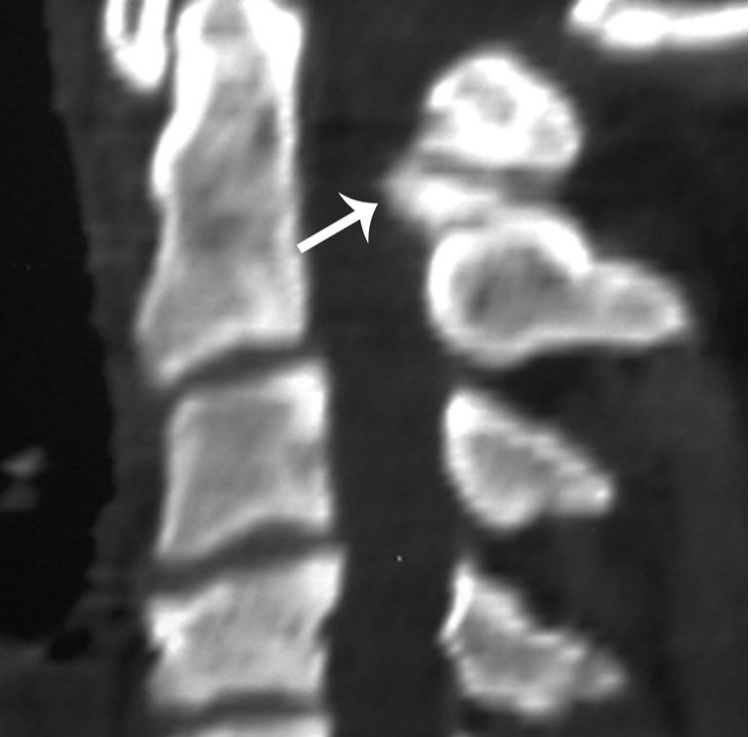
Fig 1 Axial (arrowheads) and Fig 2 sagittal CT demonstrate an expansile lesion (arrow) of the posterior arch of C1. It is contained within the cortex with no soft tissue extension. The bony margins appear smooth, homogeneous and sclerotic.

A Torg ratio [5] (ratio of canal diameter divided by vertebral body diameter on a lateral plain cervical radiograph) of 1 was measured at the C5 vertebral level. A cervical spine magnetic resonance examination (MRI) showed decreased signal intensity within the spinal cord on T1-weighted images and increased signal intensity on T2-weighted images at the level of C1 indicative of spinal cord edema and or myelomalacia. A computerized tomography examination (CT scan) demonstrated an expansile lesion involving the posterior arch of C1, with an intact overlying cortex and no soft tissue extension (Figures 1, 2).

**Figure 3:**
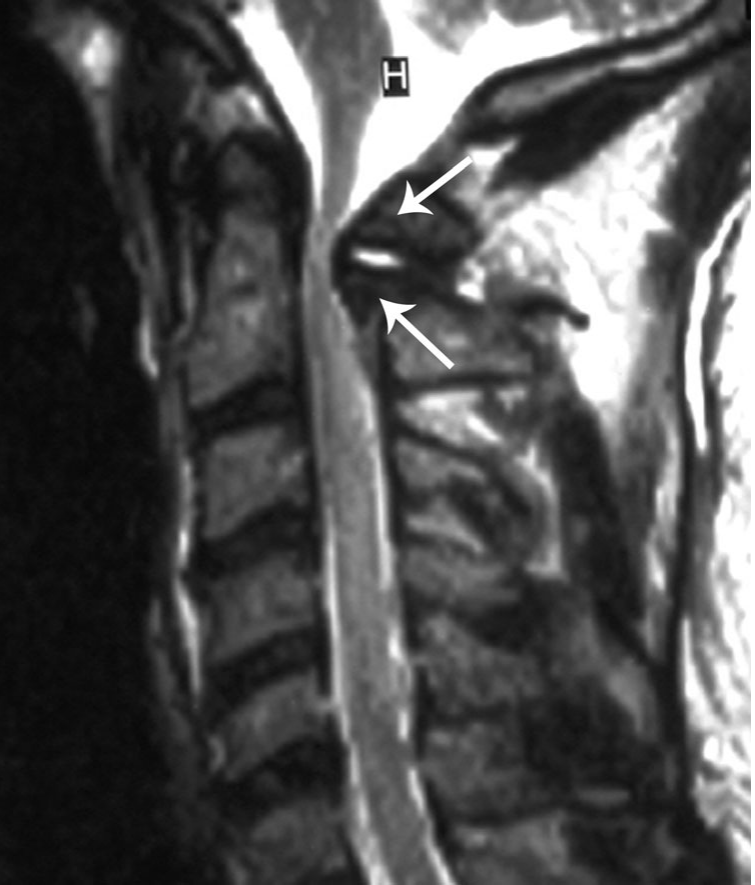
Sagittal T2 weighted MRI demonstrates an expansile lesion (arrows) of the posterior arch of C1 resulting in significant compression on the posterior thecal sac and spinal cord.

The bony margins appeared smooth, homogeneous and sclerotic, and there was a central lucency suggestive of a nidus. The expansile lesion was noted to result in significant compression on the posterior thecal sac and spinal cord at this level (Figure 3).

Radiographically, the lesion had features typical for a benign tumor such as a large osteoid osteoma or osteoblastoma, including osseous expansion, peripheral sclerosis and bony hypertrophy, internal lucency, and even suggestion of a central nidus. The lesion was greater than 1.5 cm in diameter.

**Figure 4:**
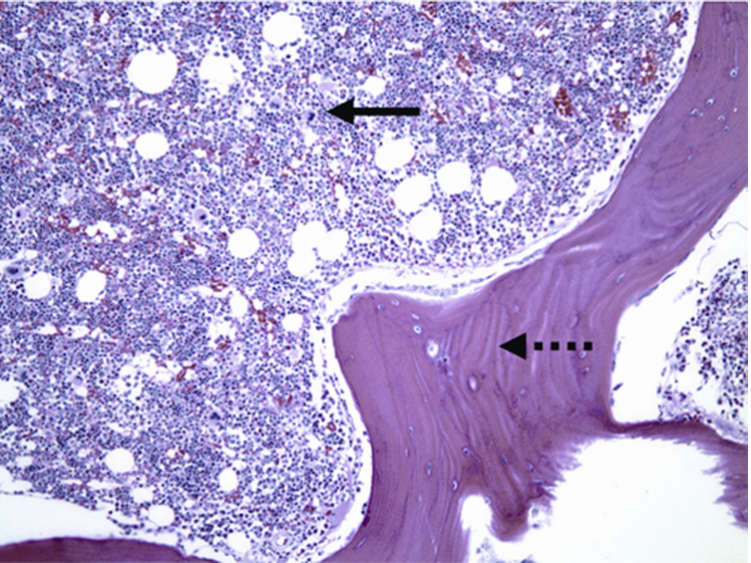
Histologically, the bony trabeculae are thickened and woven bone formation is identified at the cortical surface of the lesion. Lamellar bone formation is centrally identified. There is no evidence of nidus formation. The medullary component shows trilineage hematopoiesis and there is no definitive evidence of a neoplasm. The lesions are interpreted as reactive bone formation.

The patient subsequently underwent an en bloc resection of the posterior atlas via a posterior approach. The lamina was resected out to the margins of the C1 isthmus and vertebral arteries bilaterally. Intraoperative neuromonitoring did not reveal any abnormality prior to or following tumor resection. Due to the presence of myelomalacia and the potential for excessive neural shear stress from cervical flexion or rotation, a fusion procedure was considered, but the lack of anticipated spinal instability after surgical removal of the C1 lamina lead the patient to elect against the fusion. The surgical specimen was sent to pathology where it was noted to be consistent with simple benign osseous hypertrophy; neither consistent with an osteoid osteoma or osteoblastoma on histological analysis (Figure 4).

The patient had an uneventful postoperative course and at the latest follow-up, just over one year out of surgery, the patient was doing well without any complaints of neck discomfort or neurologic symptoms. Even so, given the lack of an intact posterior arch of C1, he was advised to refrain from contact sports due to the presence of cervical spinal cord myelomalacia.

## Discussion & conclusion

Imaging work up of developmental lesions involving the axial skeleton most frequently includes plain radiographs, followed by CT for assessment of bony matrix and MRI for evaluation of intrinsic spinal cord parenchymal changes and the potential neural compression. With some lesions, bony scintigraphy or PET scanning may be helpful to assess for metabolic activity.

This rugby player's clinical and radiographic findings suggested that the bony lesion involving the posterior elements of the cervical atlas was most compatible with an osteoblastoma, which could directly or indirectly predispose the patient to upper extremity stingers or burner. Although the surgical pathology specimen was determined to be a benign, productive osseous lesion, resection for alleviation of the mass effect on the spinal cord ultimately eliminated the patient's symptoms of the burner syndrome.

Many authors have studied athletes to determine if there are any variables or pre-existing conditions that make one susceptible to "stingers". After evaluating 165 freshman football players, Castro et al. applied the Torg ratio to their cervical imaging studies and found a relationship between the prevalance of burners in those athletes with cervical spinal stenosis. They demonstrated that college athletes with a ratio of less than 0.75 were at an increased risk for recurrent stingers, however the ratio was not related to the initial onset of a stinger [6].

In another study, Leivitz et al. reported that there is a high incidence of cervical canal stenosis in football players with recurrent burner syndrome [1].

One of the more difficult answers to determine with these injuries is an appropriate time interval beyond which an athlete can safely return to play following a traumatic cervical peripheral neuropraxia. This is a decision derived from a compilation of factors including the patient's history, severity and chronicity of symptoms, mechanism of injury, objective anatomical injury (based on physical examination or imaging abnormalities), and the athlete's recovery response [7].

Recommendations for return to play in the setting of sports related "stinger or burner" are generally based on the absence of specific structural abnormalities if imaging studies are available, and the clinical findings and include the following: complete resolution of symptoms, normalization of upper extremity strength to baseline, and normal cervical range of motion [8]. If symptoms persist, a more detailed evaluation including advanced imaging studies (MRI or CT) if not already obtained, should be performed to rule out an occult fracture, physical cord compression (herniated disk), cord parenchymal changes, instability, or structural abnormalities, before returning to play. Advanced cervical disk degeneration has been noted in athletes with chronic recurrent burner syndrome [3].

Patients with either an osteoblastoma or osteoid osteoma often present with a complaint of intermittent or constant axial spine pain, worst at night, and responsive to aspirin or NSAIDs. In addition to axial pain, neural compression by the tumor may cause clinical manifestations of myelopathy, radiculopathy or a combination of these [9]. As a result, these tumors should generally be considered, although not likely, in the differential diagnosis of young patients with complaints of persistent or recurrent axial pain and radicular symptoms.

While an osteoid osteoma or osteoblastoma involving the spine can often be diagnosed with radiographs, advanced imaging including MRI and/or CT is generally indicated to define the nature and extent of soft tissue involvement or compromise. For example, Raskas et al. reported a 57% incidence of epidural invasion in patients with a documented osteoblastoma [10].

In summary, the burner syndrome is most often a benign condition commonly experienced by athletes participating in collision sports. Symptoms are typically self-limited, resolving within hours to days. In cases where symptoms fail to resolve, or the patient experiences several recurrent episodes, further clinical and imaging investigation should be performed to exclude possible lesions of the cervical spine.

Return to play is predicated on the absence of intrinsic cord abnormalities, instability or symptoms of neck pain, lack of cervical range of motion, or neurologic symptoms [7,8].

We conclude that complete en bloc resection of the benign lesion in our case, which turned out to be hypertrophic bone, revealed very good clinical results.
